# Editorial: Hepato-pancreato-biliary surgery: innovations, genomics, and prognostic management

**DOI:** 10.3389/fsurg.2024.1386870

**Published:** 2024-03-01

**Authors:** Ahmad Mahamid

**Affiliations:** ^1^ Department of Surgery, Carmel Medical Center, Haifa, Israel; ^2^ The Ruth and Bruce Rappaport Faculty of Medicine, Technion-Israel Institute of Technology, Haifa, Israel

**Keywords:** hepato-pancreato-biliary surgery, diagnostic and surgical technologies, surgical and diagnostical technological innovations, multidisciplinary peri-operative management, prognostic clinical, pathological, molecular genomics

**Editorial on the Research Topic** Hepato-pancreato-biliary surgery: innovations, genomics, and prognostic management

Hepato-Pancreato-Biliary surgery stands as the cornerstone of treatment for both primary and secondary tumors, offering a vital pathway to prolonged survival. Over the past few decades, remarkable strides have been made in enhancing the short and long-term outcomes within this domain. The collective efforts in multidisciplinary peri-operative care, coupled with advancements in diagnostic and surgical technologies, have significantly reshaped the landscape of Hepato-Pancreato-Biliary surgery.

Cholecystectomy, has evolved over time. Initially performed as an open surgery involving a large incision, it has transitioned to minimally invasive laparoscopic techniques. Advanced technologies like robotic-assisted surgery have further refined the procedure. Laparoscopic cholecystectomy is considered the gold standard for gallbladder removal due to its minimally invasive nature, leading to faster recovery, reduced post-operative pain, and shorter hospital stays compared to open surgery. but it also can lead to severe complications like bile duct injury (BDI) (1, 2).

Vu et al., in their research found that the majority of BDIs are type (A) and diagnosed postoperatively. Endoscopic retrograde cholangiopancreatography (ERCP) was effective for the diagnosis and treatment of this type injuries.

ERCP has evolved significantly since its introduction. Initially a diagnostic tool, it has developed into a therapeutic procedure for managing various pancreaticobiliary disorders. Advances include improved imaging technologies, better endoscopic instruments, and techniques like endoscopic ultrasound (EUS) and cholangioscopy, enhancing the precision and effectiveness of interventions.

Uhe et al., in their case report showed that the diagnosis of entero-biliary fistula can be made by ERCP, which not only allow to visualize the fistula orifice and tract through the injection of contrast, but also allow definitive treatment with sphincterotomy and the implantation of a covered self-expandable metal stent in the common bile duct.

Complications associated with ERCP encompass pancreatitis, hemorrhage, infection, perforation, and adverse reactions to sedatives or contrast agents. Factors such as advanced age, comorbidities, and procedural complexity can elevate the risk profile for these adverse events. Lee et al., reported the long-term sequalae of a rare case of a patient with a retained fractured end of the basket, used in ERCP, on the head of the pancreas, resulting in chronic pancreatitis with gross pancreatic atrophy, pancreatic duct dilatation, and early-onset diabetes mellitus.

Duodenal diverticula (DD) is one of the most common digestive tract diverticula (3), that might increase the complexity of the ERCP procedure. Ren et al., conducted a retrospective study to explore the clinical characteristics of patients with symptomatic DD and to generalize how to make appropriate treatment choices for this group of patients. They found that Patients with DD ≥1 cm or located periampullary were more likely to be symptomatic. The size of the DD and the combination of specific biliary comorbidities may have an impact on the choice of treatment modality. Although most patients with symptomatic DD can be treated conservatively only, surgical treatment is also a safe and effective approach when the appropriate procedure is chosen.

However, when planning for surgical intervention, it's very crucial to be familiar with the standard human body anatomy and its variants. The anatomy of the hepato-pancreatico-biliary system is intricate, with structures such as the liver, pancreas, gallbladder, and bile ducts closely interconnected. This complexity presents challenges during surgical interventions, as any procedure in this area requires a precise understanding of the individual anatomy, as well as the interactions between these organs. Surgeons specializing in HPB surgery must navigate these structures with extreme care to minimize the risk of complications and ensure optimal patient outcomes.

Prepancreatic postduodenal portal vein (PPPV) is a rare congenital variant of portal vein system, with only 16 cases reported in the literature, of them five underwent pancreaticoduodenectomy (PD) (4). Tang et al., in their case report concluded that PPPV is a rare portal vein anomaly that can be easily diagnosed by preoperative imaging. Awareness of PPPV may prevent injury or dissection of this variant vessel, which could result in fatal hemorrhage and liver ischemia. Preoperative diagnosis of PPPV is essential for surgical planning, including the selection of approach and site of dissection of the pancreatic neck, and the choice of resection/reconstruction or isolation depending on the PPPV morphology.

In this Special Issue, we aimed to delve into the cutting-edge developments that define the current state of Hepato-Pancreato-Biliary surgery. Our focus encompasses the latest surgical and diagnostic technological breakthroughs in the field of Hepato-Pancreato-Biliary surgery. A field that still rapidly evolving.
